# Supplementary material for: Establishment of a locally adaptive allele in multidimensional continuous space
Source: G3 (Bethesda). 2024 Nov 15;15(1):jkae266. doi: 10.1093/g3journal/jkae266 (PMC11708235; doi:10.1093/g3journal/jkae266)
Supplement: jkae266_Supplementary_Data [file jkae266_supplementary_data.zip › Supplemental_Material_G3-2024-405482.pdf]

Supplementary materials for “Establishment of a locally adaptive allele in multidimensional continuous space”

Takahiro Sakamoto

November 8, 2024

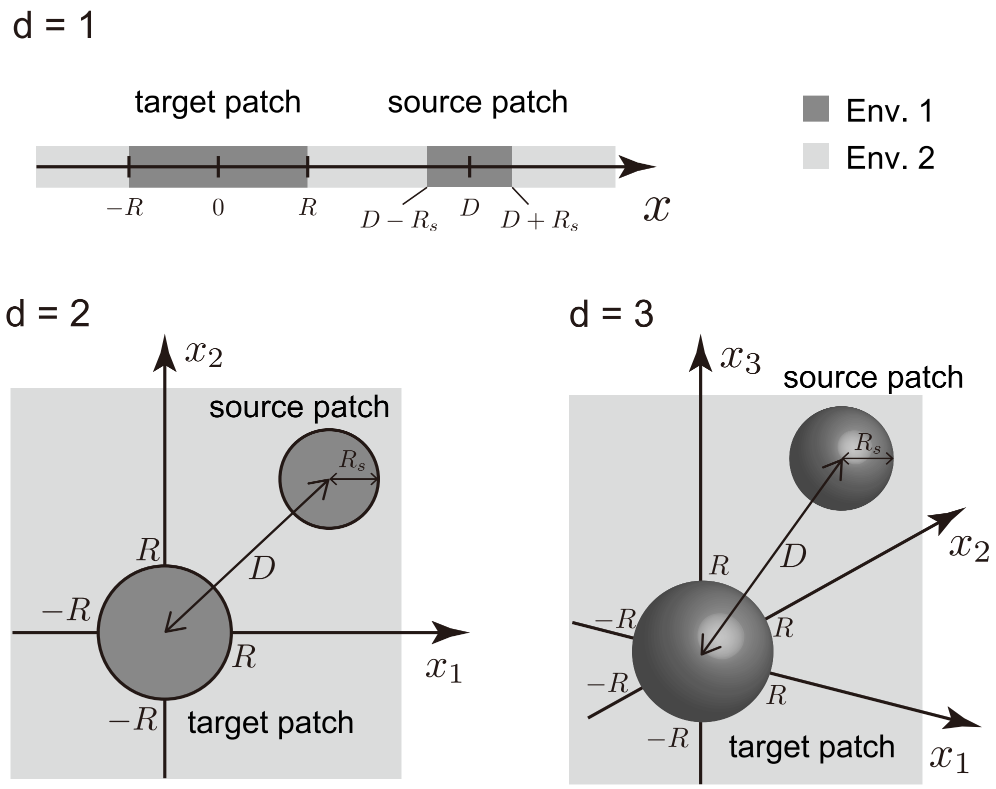

Figure S1: Illustration of the two-patch model.

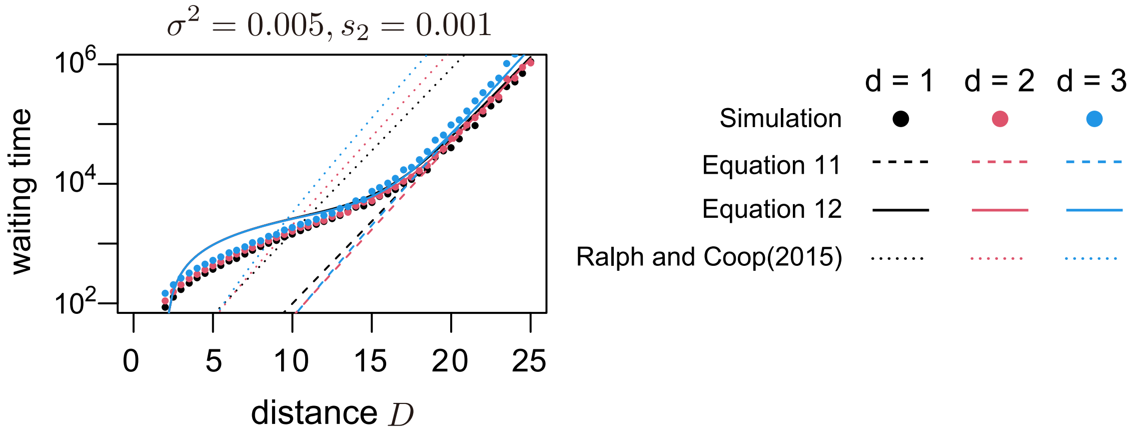

Figure S2: A version of the panel D of Figure 6 in the main text assuming a wider range of D.

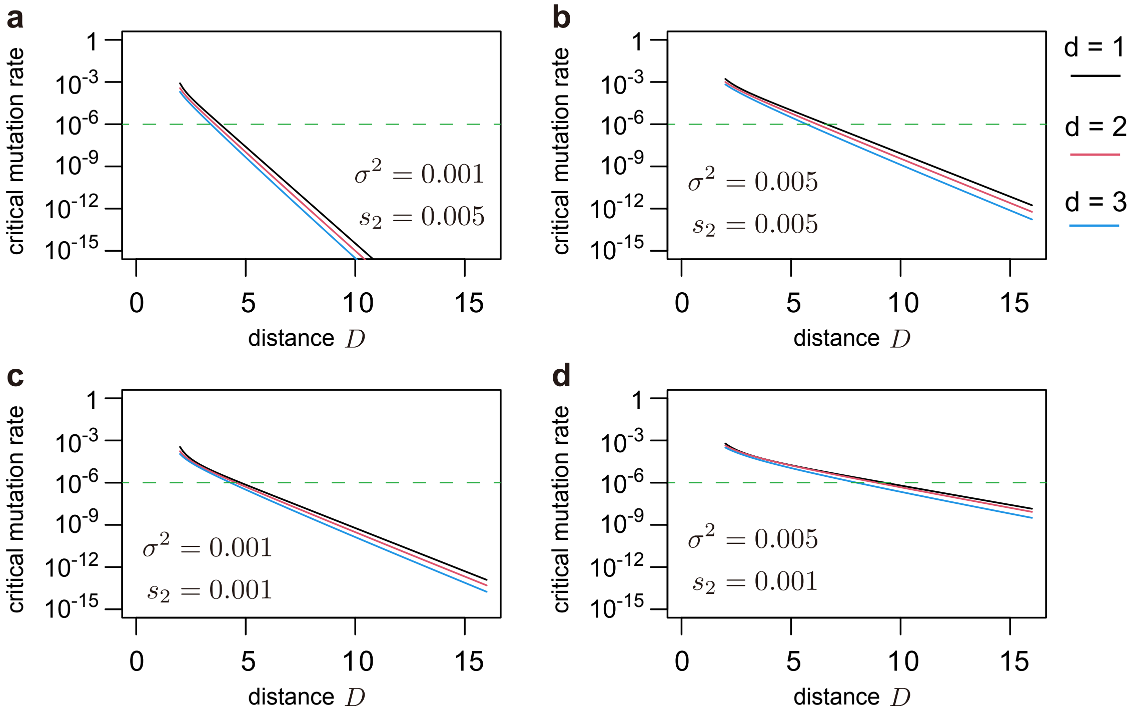

Figure S3: Mutation rate at which the relative contributions of mutation and migration are balanced ($\lambda_{\text{mut}} = \lambda_{\text{mig}}$). Green dashed lines are drawn at $\mu = 10^{-6}$. Parameter values mirror those given in Figure 6 in the main text. In each panel, following parameters were assumed: (a) $\sigma^2 = 0.001$ and $s_2 = 0.005$, (b) $\sigma^2 = 0.005$ and $s_2 = 0.005$, (c) $\sigma^2 = 0.001$ and $s_2 = 0.001$, and (d) $\sigma^2 = 0.005$ and $s_2 = 0.001$.

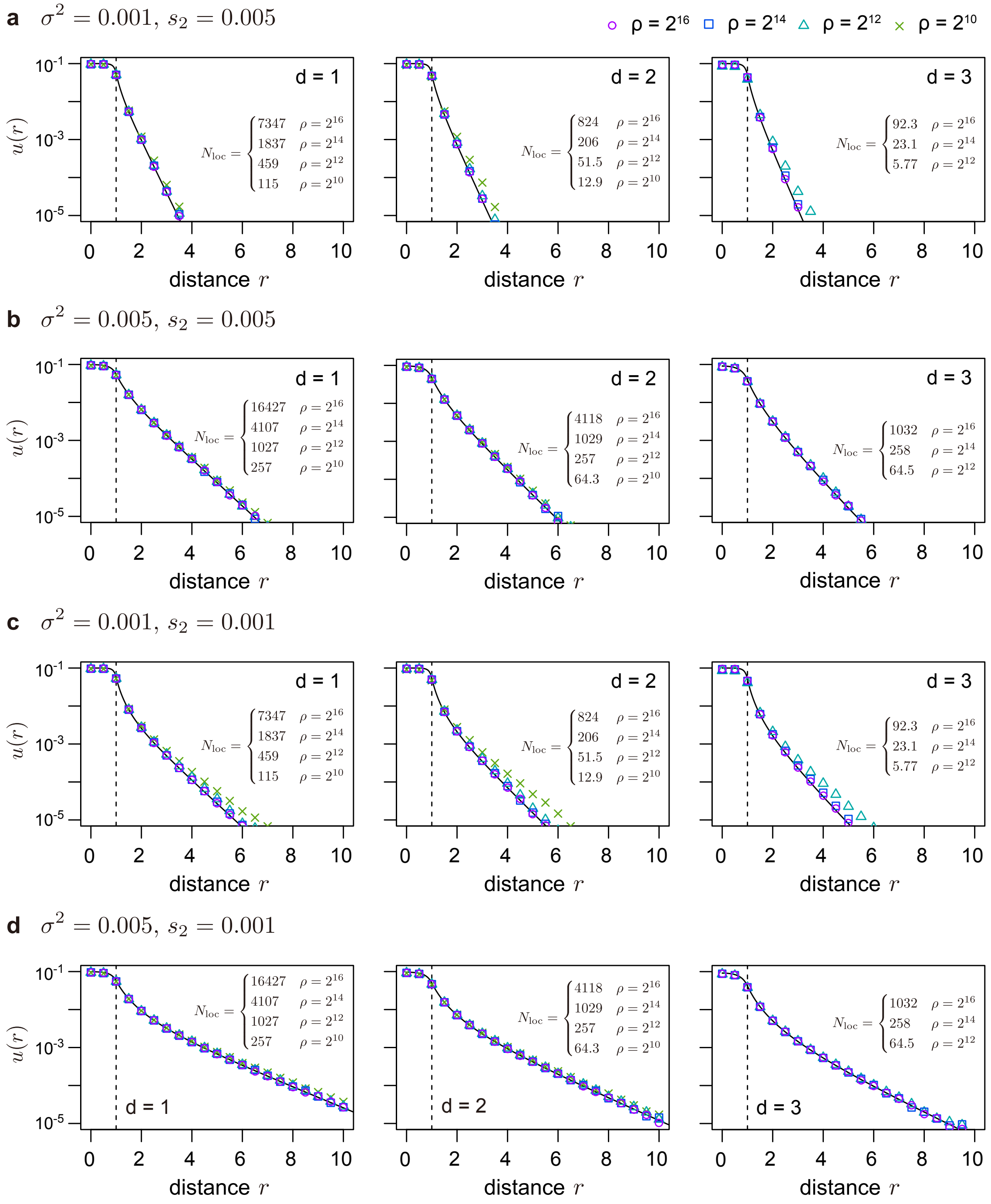

Figure S4: Establishment probability a locally adaptive mutation in lower population densities. Parameter values mirror those given in Figure 2 in the main text except for population density. In each row, following parameters were assumed: (a) $\sigma^2 = 0.001$ and $s_2 = 0.005$, (b) $\sigma^2 = 0.005$ and $s_2 = 0.005$, (c) $\sigma^2 = 0.001$ and $s_2 = 0.001$, and (d) $\sigma^2 = 0.005$ and $s_2 = 0.001$. For each parameter set, 10^7 simulation replicates were run. Vertical dashed lines are drawn at $r = R$, marking the boundary between the two environments.

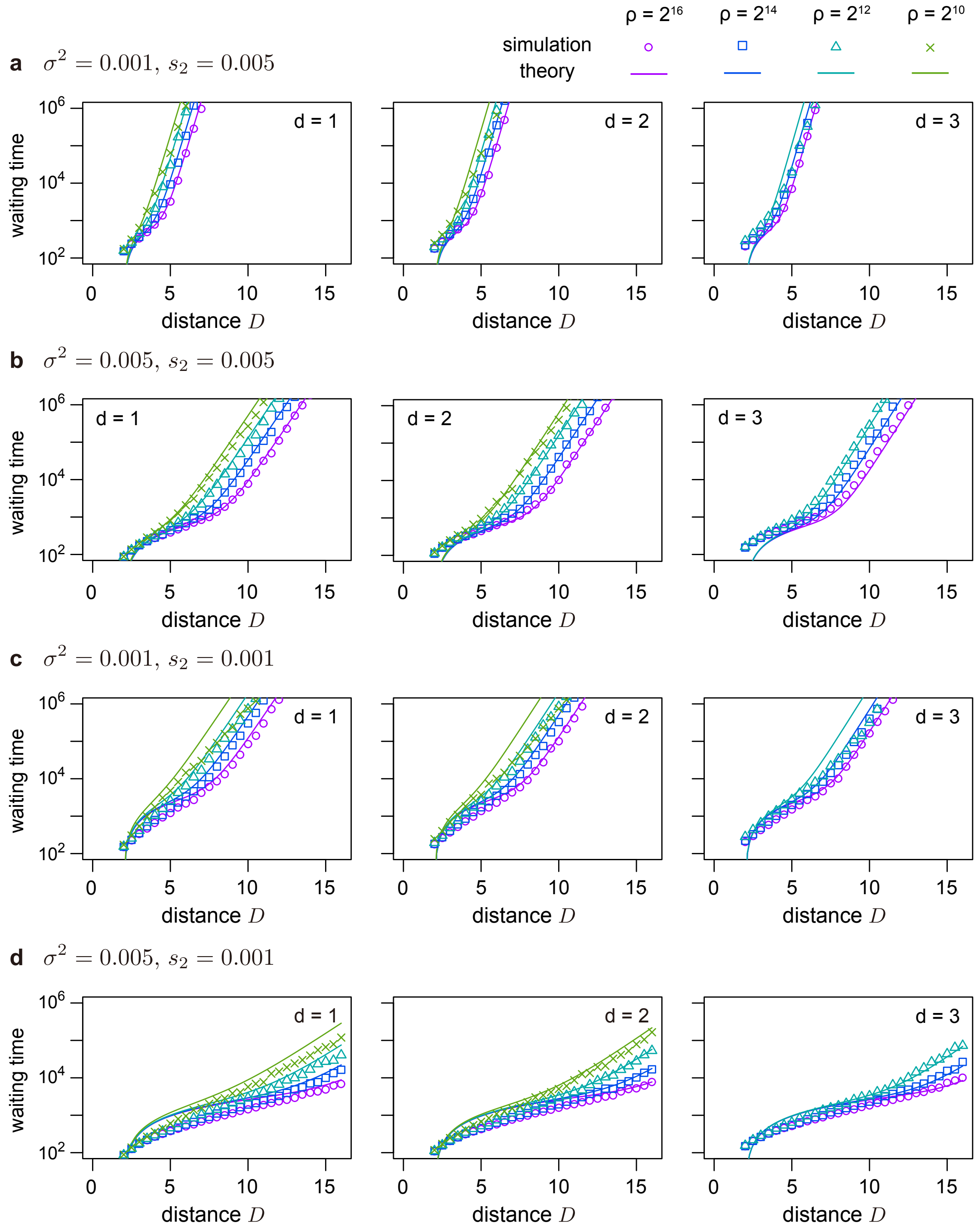

Figure S5: Waiting time until local adaptation through immigration in lower population densities. Parameter values mirror those given in Figure 6 in the main text except for population density. In each row, following parameters were assumed: (a) $\sigma^2 = 0.001$ and $s_2 = 0.005$, (b) $\sigma^2 = 0.005$ and $s_2 = 0.005$, (c) $\sigma^2 = 0.001$ and $s_2 = 0.001$, and (d) $\sigma^2 = 0.005$ and $s_2 = 0.001$. Each color represents the results for different population density. For each parameter set, 100 simulation replicates were run.
